# The circular RNA circCPE regulates myoblast development by sponging miR-138

**DOI:** 10.1186/s40104-021-00618-7

**Published:** 2021-09-08

**Authors:** Wenxiu Ru, Ao Qi, Xuemei Shen, Binglin Yue, Xiaoyan Zhang, Jian Wang, Hui Cao, Hong Chen

**Affiliations:** 1 Key laboratory of Animal Genetics, Breeding and Reproduction of Shaanxi Province, College of Animal Science and Technology, Northwest A&F University, Yangling, 712100 Shaanxi China; 2 Shaanxi Kingbull Livestock co.,LTD, Yangling, 712100 Shaanxi China

**Keywords:** Apoptosis, Bovine, CircRNA, Differentiation, MiR-138, Proliferation

## Abstract

**Background:**

Skeletal muscle development, a long-term and complex process, is controlled by a set of the myogenic genes. Circular RNAs (circRNAs), a class of noncoding RNA, have been shown to regulate various biological processes. Recent studies indicate circRNAs may be involved in myogenesis, but the role and regulatory mechanism of circRNAs in myogenesis is largely unknown. In the present study, circCPE was firstly found to promote the bovine myoblast proliferation and inhibit cell apoptosis and differentiation by influencing the expression of *FOXC1* in a miR138-mediated manner. And *in vivo* experiments revealed that overexpression of circCPE attenuates skeletal muscle regeneration.

**Results:**

We identified a novel circular RNA circCPE by analyzing circRNAs sequencing data of bovine muscle tissue. Sequencing verification, RNase R treatment and Actinomycin D treatment confirmed the circular nature of circCPE in bovine muscle. Functional assays showed that overexpression of circCPE could inhibit bovine myoblast apoptosis and differentiation, as well as facilitate cell proliferation. Moreover, *in vivo* experiments revealed that overexpression of circCPE attenuates skeletal muscle regeneration. In consideration of circRNA action as miRNAs sponge, we found that circCPE harbors miR-138 binding sites and absorbed miR-138. Mechanistically, the rescue experiments showed that the overexpression of circCPE can counteract the inhibitory effect of miR-138 on the cell proliferation and the accelerated effects on the differentiation and apoptosis. Subsequently, we found that circCPE sequester the inhibitory effect of miR-138 on *FOXC1* so as to involve in myogenesis.

**Conclusions:**

Collectively, we constructed a novel circCPE/miR-138/*FOXC1* regulatory network in bovine myogenesis, which further provide stronger evidence that circRNA involved in muscle development acting as miRNA sponge.

**Supplementary Information:**

The online version contains supplementary material available at 10.1186/s40104-021-00618-7.

## Background

Circular RNAs (circRNAs), a class of covalently closed-loop RNAs, are generated by back- splicing of exons in protein-coding genes [1]. The advance of RNA sequencing (RNA-seq) method and bioinformatics technology has uncovered abundant circRNAs, which are widely distributed in metazoans. Because the efficiency of back-splicing is not high, there are lower expression levels of circRNAs [2]. However once produced, circRNAs are generally stable which possess RNaseR resistance and longer half-lives [3, 4]. Frequently the majority of circRNAs are primarily localized in the cytoplasm and circRNAs containing introns are left in the nucleus [5, 6], which suggested the diverse functions of circRNAs. Recent studies have shown that circRNAs tend to have different functions including modulating transcription and splicing, serving as miRNA or protein sponges, acting as protein scaffolds, and synthetizing polypeptides [1]. There has been extensive study of circRNAs function as competing endogenous RNAs (ceRNAs), which can sponge miRNAs thus prevent them from suppressing their target mRNAs in the cytoplasm [7]. Of all the reported circRNAs in this category, the most striking example is ciRS-7 (also known as *CDR1as*) and the testis specific circSry. CiRS-7 contains more than 70 conserved target sites for miR-7 and circSry contains 16 target sites for miR-138 [7, 8]. An increasing number of circRNAs has been implicated in a variety of biological processes, involving organism development, cell proliferation and transformation, human diseases and so on [1].

Skeletal muscle as the main tissue in the body participated in movement and metabolism [9]. For livestock, the development of skeletal muscle directly impacts the meat quality. Skeletal muscle development, a long-term and complex process, mainly experience myoblast proliferation and differentiation, fuse into myotubes, and finally muscle fibers formation [10]. To date, several studies have uncovered important regulatory mechanisms by which the myogenic regulatory factors (MRFs), signal transduction pathway, and non-coding RNAs (ncRNAs) control the specification and the differentiation of skeletal muscle development [11]. The recent findings on the mechanisms used by non-coding RNAs to regulate skeletal muscle formation have become more extensive [12].

MicroRNAs (miRNAs) are endogenous ncRNAs about 23 nt that play important regulatory roles by binding the 3′ UTR of mRNAs to repress their posttranscriptional expression or translation [13]. There are many reported roles of miRNA in skeletal muscle development. Such as miR-1, miR-133, miR-206, and miR-486 could target some myogenic regulatory factors (MRF) to regulate skeletal muscle development [11, 14–16]. Long non-coding RNAs (lncRNAs) are longer than 200 nt and also act as ceRNAs to regulate transcription by decoying miRNAs [17]. Recently, studies have revealed the important role of lncRNAs on myogenesis, such as lncMD, lncH19, and lncMDNCR was required for primary myoblast differentiation [18–20]. Additionally, recent studies provide evidence that circRNAs also involved in skeletal muscle development. CircZfp609 was identified to repress myogenic differentiation by sponging miR-194-5p in the mouse [21]. A well-known CDR1as was showed to promote muscle differentiation by relieves the downregulation of *IGF1R* (insulin like growth factor 1 receptor) caused by miR-7 [22]. Moreover, our previous studies have also identified some circRNAs acted as miRNAs decoy to regulate bovine myoblast development, such as circFUT10, circHUWE1, circINSR, and so on [23–25]. Overall, research on circRNAs in skeletal muscle development is updating and there remains more work to explore the functions of circRNAs.

In this study, we noticed the differential expression of circCPE in fetal and adult bovine muscle tissue by analyzing circRNAs sequencing data of bovine muscle tissue from NCBI (Accession ID: GSE87908). CircCPE generated from second and third exons of carboxypeptidase E (*CPE*), which was firstly covered in this study. Our results indicated that circCPE promoted the bovine myoblast proliferation and inhibited cell apoptosis and differentiation. Moreover, we found circCPE might be involved in myogenesis by influencing the expression of *FOXC1* in a miR138-mediated manner. These results provide stronger evidence that circRNA involved in muscle development acting as miRNA sponge.

## Materials and methods

### Sample preparation

Animal samples used in this study were approved by the Animal the Ethics Committee of Northwest A&F University. We collected muscle samples at three developmental states (fetal, 90 d; calf, 6 months; and adult, 24 months) of Qinchuan cattle from a local livestock farm of Xi’an (Shannxi, China). Other tissue samples, including heart, liver, spleen, lung, kidney were obtained at the fetal stage (90 d), and adipose tissues were obtained at the adult stage (24 months). All samples were immediately snap-frozen in liquid nitrogen after surgical removal and stored at − 80 °C freezer until use.

### Establishment of muscle injury animal model

Mice (C57BL/6) were purchased from the Fourth Military Medical University (Xi’an, China). Animal care and study protocols were approved by the Animal Care Commission of the College of Veterinary Medicine, Northwest A&F University. Cardiotoxin (CTX; Whiga) was dissolved in sterile saline to a final concentration of 10 μmol/L and injected with 50 μL of CTX into the Right TA muscles of ~ 6-week-old mice to induce skeletal muscle damage. TA tissue samples were collected at 0, 3 d after muscle injury. The samples were fixed for haematoxylin-eosin (HE) staining.

### Cell culture

Bovine primary myoblasts were isolated from longissimus muscle at fetal state as described in previous studies [26]. The brief steps are as follows. The muscle tissue was cut to pieces and digested with the concentration 2 mg/mL of collagenase I at 37 °C for about 2 h. Then, the digested muscle tissue was filtered through a 200-mesh filter. The filtrate was washed and centrifuged three times with PBS at a low speed. Finally, the cell sedimentation was resuspended with growth medium containing 20% fetal bovine serum (FBS, Gibco) and 1% penicillin-streptomycin (HyClone, USA), and were cultured at 37 °C with 5% CO_2_. HEK-293 T cells were cultured in DMEM with 10% FBS and 1% penicillin-streptomycin. For myoblast differentiation, myoblasts were cultured in differentiation medium (DMEM containing 2% horse serum and 1% penicillin-streptomycin).

### Vector construction and transfection

To overexpress circCPE, a fragment of 301 bp of cDNA was cloned into pcDNA2.1 vector (Geneseed, China). We respectively structured psiCHECK2-cirCPE (Promega, Madison, WI, USA) with full length of circCPE and psiCHECK2-cirCPE- Mut with mutational sites pairing to the miR-138. The miR-138 mimics was synthesized by RiboBio (Guangzhou, China). The reverse complement repeats of miR-138 seed region were synthesized and inserted into psiCHECK-2 vector to constitute miR-138 biosensor. Consistently, we generated the vectors of psiCHECK-FOXC1-WT/Mut. All vectors and mimic were followed the procedure according to the manufacturer’s instructions to transfection (R0531 Thermo Fisher Scientific, USA).

To experimentally overexpression of circCPE *in viv*o, we mixed 6.25 μg pcDNA2.1- circCPE plasmid with 12.5 μL glucose solution to make reagent A, and reagent B was mixed by 12.5 μL Entranster™-in vivo (Engreen, Beijing, China) with 12.5 μL glucose solution. In a final, reagent A and reagent B mixed thoroughly to form 50 μL mixture. The mixture was injected into 3 mice TA muscles to serve as treatment group and another 3 mice injected a pcDNA2.1 plasmid glucose solution to serve as the control group. Injection time was 12, 48, and 96 h after CTX treatment and samples were collected at 3, 5, and 7 d.

### RNA extraction and real-time qRT-PCR analysis

Total RNA was isolated from tissues and cells using TRIzol reagent (Takara, Japan) according to the manufacturer’s protocol. cDNA was synthesized using a RT reagent kit (Takara, Japan) and qRT-PCR for circRNA and mRNA was performed on the CFX connect real-time system (Bio-Rad, USA) using a SYBR Premix Ex Taq II kit (Takara, Japan). *β-actin* and *U6* were respectively employed as the internal control for mRNA, circRNA, and miRNA detection. Each sample was replicated three times and quantitation data was calculated by comparing Ct values. All primers are listed in Table S1.

### RNase R and Actinomycin D treatment

Total RNA (5 μg) from tissues was digested with RNase R (Epicentre, Madison, WI, USA) at 37 °C for 2 h and postdigestive RNA (1 μg) was used to synthesize cDNA. Actinomycin D (Applied Biological Materials Inc., CAN) was added to the medium of cells for testing the half-life of circCPE and linear mRNA. The final concentration of Actinomycin D is 2 μg/mL for testing the half-life of circCPE and linear mRNA.

### RNA-FISH assay

RNA Fluorescent in situ hybridization (RNA-FISH) was performed on bovine primary myoblasts using the probe that can specifically recognize the back-splicing junction of circCPE (RiboBio, Guangzhou, China). Cells were cultured in the 6-well plates containing cover glass to 70% ~ 80% confluence, then were fixed with in situ hybridization fixative. After penetrated by 0.1% Triton X-100, the cells were incubated with the circCPE probes overnight at 37 °C. Nuclei were stained with DAPI. Laser confocal microscopy was used to acquire the images (Nikon, Tokyo, Japan).

### Western blotting assay

Cells were lysed with radio immunoprecipitation assay buffer (RIPA) containing 1 mmol/L PMSF (Solarbio, China). Protein extractions were separated by 12% SDS-PAGE gels and transferred to polyvinylidene fluoride (PVDF) membranes (Millipore, Germany). The membranes were incubated overnight at 4 °C with primary antibodies specific for anti-β-actin, anti-CDK2, anti-CyclinD1, anti-CyclinE, anti-PCNA, and anti-P53 (1:1,000; Wanleibio), anti-Bcl-2, anti-Bax, anti-Caspase9, anti-FOXC1, and anti-MyoD1 (1:1,000; Abways Technology), anti-MyoG, (1:1,000; Abcam, Cambridge, England). After incubation with secondary antibodies, signals were detected using a chemiluminescence system (Bio-Rad, USA).

### Cell proliferation assay

For the cell proliferation assay, the transfected myoblasts cultured in 96-well plate were treated with 10 μL of CCK-8 (Solarbio, China) and each sample was replicated eight times. After incubation at 37 °C for 2 h, the absorbance of each sample was detected at 450 nm using a microplate reader (BioTek, USA). Cell proliferation was also detected by EdU assay kit (RiboBio, Guangzhou, China) following the manufacturer’s protocol. Images were finally obtained by fluorescence microscopy (DM5000B; Leica Microsystems, Germany).

### Cell cycle and apoptosis assays

For the cell cycle analysis, the myoblasts were cultured in 60-mm plates and stained with propidium iodide (PI) after transfection for 24 h. Then using flow cytometry (BD Biosciences, USA), the ratios of cells in the G0/G1, S, G2 phases were counted and compared. To detect cell apoptosis, the transfected myoblasts were stained using an annexin V-APC/7AAD apoptosis kit (eBiosciences, USA) and analyzed using flow cytometry. The early apoptotic cells and late apoptotic cells were counted and compared. Each sample was independently performed in triplicate.

### Immunofluorescence staining

Myoblasts were cultured in differentiation medium (DMEM containing 2% horse serum and 1% penicillin-streptomycin) for 5 d. Then cells were washed three times with PBS and fixation in 4% paraformaldehyde for 30 min and permeabilized for 15 min using 0.5% Triton X-100. After closed with 5% BSA, the cells were incubated at 4 °C overnight with antibody-MyHC (1:250; GTX20015, GeneTex, USA). We used the corresponding secondary antibody goat anti-mouse IgG (H&L)-Alexa Fluor (1:500; RS3608, Immunoway, USA) to incubate cells at room temperature for two hours. Nuclei were stained with DAPI. Finally, the cells were washed and observed with fluorescence microscope (DM5000B; Leica Microsystems, Germany). The differentiation index was calculated as the percentage of nuclei in MyHC positive cells.

### Luciferase reporter assay

HEK293T cells were seeded in 96-well plates and co-transfected with plasmids including pCD2.1-circCPE, psiCHECK2-cirCPE, psiCHECK2-cirCPE-Mut, miR-138 biosensor, psiCHECK-FOXC1-WT/Mut, and miR-138 mimics using Troubfect (R0531 Thermo Fisher Scientific, USA). After transfection for 24 h, firefly and renilla luciferase activities were detected using Dual-Luciferase Reporter Assay Kit (E2920, Promega, Fitchburg, WI, USA) according to the manufacturer’s instructions. Finally, ratios of renilla to firefly luciferase were calculated and each assay was repeated in 8 independent experiments.

### RIP assay

RNA binding protein immunoprecipitation (RIP) assay was performed using Magna RIP RNA-binding protein immunoprecipitation kit (Millipore, USA) according to the manufacturer’s instructions. Myoblasts were harvested and lysed using RIP lysis buffer. Then the lysates were respectively incubated with beads that coated with Ago2 (Abcam, England) antibody and mouse IgG antibody (Millipore, USA) at 4°C overnight. The beads were washed with lysis buffer and the bound RNA in the magnetic beads was extracted using Trizol reagent and the abundance of circCPE and miR-138 analyzed by qRT-PCR assay.

### Statistical analysis

All date is showed as mean ± SEM based. The analyses were performed with SPSS 22.0 (SPSS, USA). Data differences between two groups were analyzed by the Student’s test and one-way analysis of variance (ANOVA) was used to compare three or more groups. An asterisk was considered to be *P*-value < 0.05 and two asterisks represent *P*-value < 0.01.

## Results

### Characterization of bovine circCPE

**Fig. 1:**
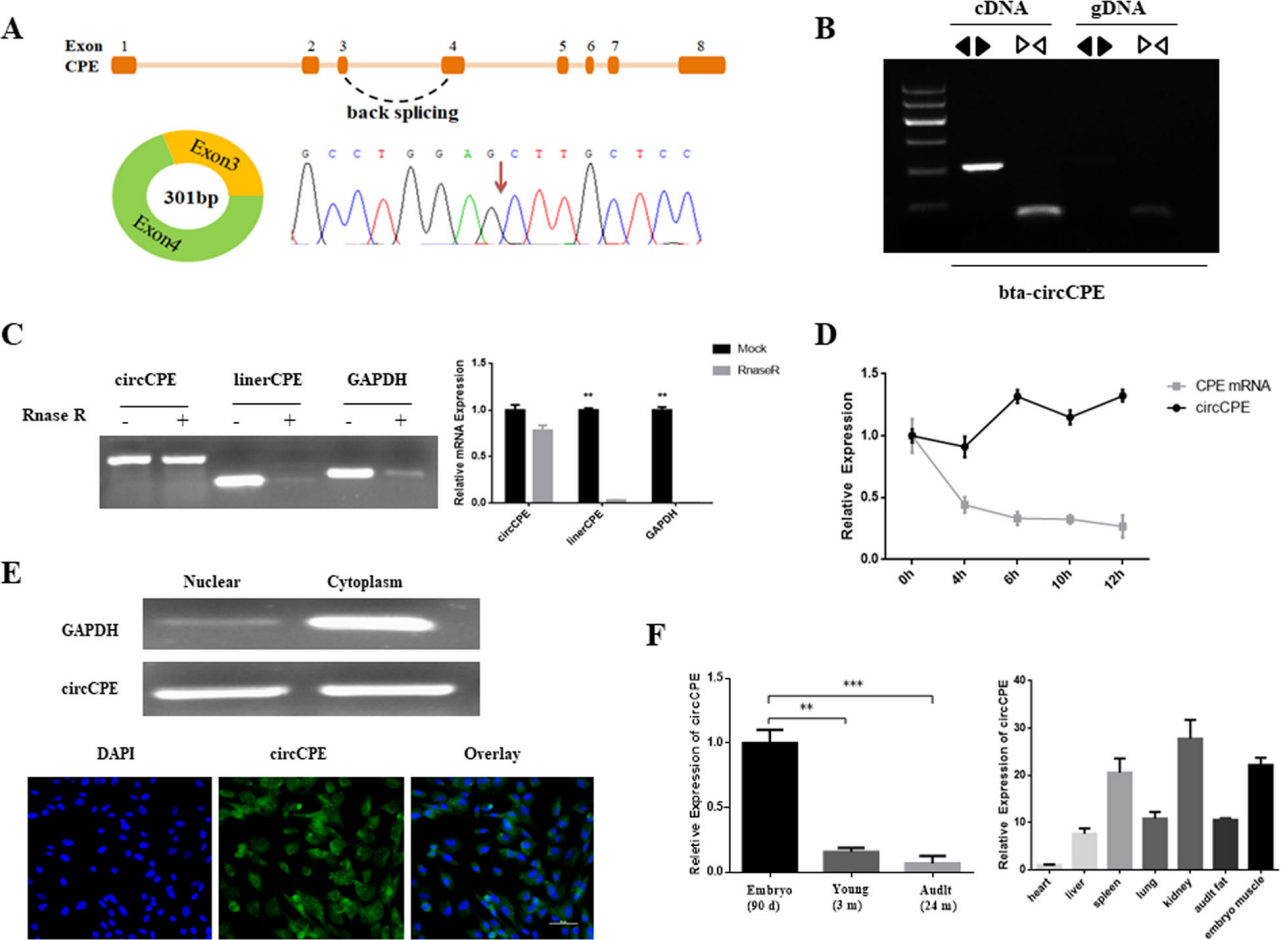
Characterization of bovine CircCPE. (**A**) Schematic showing the circularization of CPE exon 3 and exon 4 forming circCPE. The back- splicing junction of circCPE was confirmed by Sanger sequencing. (**B**) The existence of circCPE was verified with PCR and agarose gel electrophoresis assay using divergent primers and convergent primers. (**C**) Resistance to RNase R of circCPE was tested using agarose gel electrophoresis and qRT-PCR assay (**D**) qRT-PCR for the abundance of circCPE and CPE mRNA in myoblasts treated with Actinomycin D at the indicated time points, and the results indicated that circCPE was more stable than linear CPE. (**E**) Cellular localization analysis and the RNA-FISH assay showed that circCPE is localized both in the cytoplasm and nuclear. *GAPDH* is positive control for cytoplasmic fraction. Blue indicates nuclei stained with DAPI; green indicates the RNA probe that recognizes circCPE. (**F**) Expression profile of circCPE in muscle tissues from fetal to adult cattle and in different tissues of fetal cattle. Data are presented as means ± SEM. *P* < 0.05, *P* < 0.01

We selected differentially expressed circCPE derived from second and third exons of carboxypeptidase E (*CPE*) in fetal and adult bovine muscle tissue from our previous results (Text S1) [27]. The presence of circCPE was first verified with RT-PCR using divergent primers and convergent primers, and the back- splicing junction was identified by Sanger sequencing (Fig. 1A, B). The results showed that circCPE was only amplified in cDNA using divergent primers, but not in gDNA (genomic DNA) (Fig. 1B). We further confirmed that the circCPE can resistant to RNase R and the levels of linear transcript and *GAPDH* were used to prove the efficacy of RNase R treatment (Fig. 1C). To assess the stability, we found that the circCPE is more stable than linear transcript after treatment with Actinomycin D in bovine myoblast (Fig. 1D). These results confirmed that the circular nature of circCPE in bovine muscle. Cellular localization analysis indicated that circCPE localized both in the cytoplasm and nuclear using *GAPDH* as positive control for cytoplasmic fraction, and the RNA-FISH assay using a specific RNA probe for circCPE is also confirmed (Fig. 1E), which imply circCPE may possess multiple functions. Next, we quantified the level of the circCPE in different stages of bovine muscle and demonstrated that circCPE was significantly higher in embryonic muscle, which consistent with the sequencing data (Fig. 1F). In addition, circCPE exhibits diverse expression patterns across a variety of bovine tissues (Fig. 1F).

### CircCPE promotes the proliferation of myoblasts

**Fig. 2:**
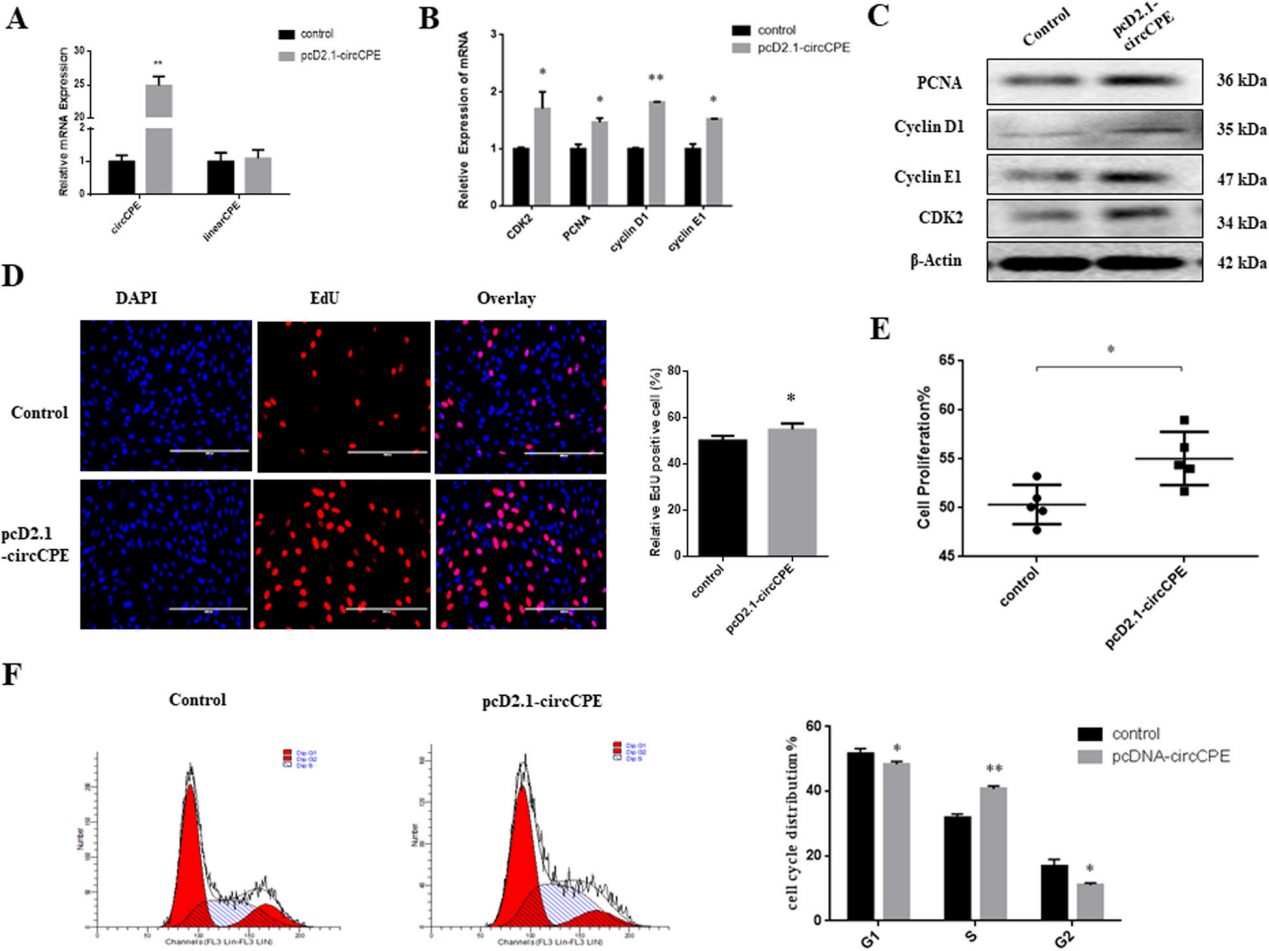
Overexpression of circCPE promotes the proliferation of Myoblasts. (**A**) circCPE was successfully overexpress but had no effect on the linearCPE mRNA. (**B** and **C**) The expression of *CDK2*, *PCNA*, *Cyclin D1*, and *Cyclin E* were detected by real-time qPCR and western blots after transfection with pcD2.1-circCPE vector. (**D** and **E**) EdU (Scale bars, 2000 μm) and CCK-8 assays were used to measure the ability of proliferation. (**F**) The cell cycle distribution was evaluated using a flow cytometry assay. Values are means ± SEM for three individuals. *P* < 0.05, *P* < 0.01

To explore the role of circCPE in bovine muscle development, we structured the overexpression vector of the pCD2.1-circCPE, and it successfully overexpressed circCPE but had no effect on the linear CPE mRNA in primary bovine myoblasts after transfection for 24 h (Fig. 2A). Our results demonstrated that overexpression of circCPE significantly increased the expression of marker genes of proliferation including *PCNA, CDK2, cyclin D* and *cyclin E* using qRT-PCR and western blot analysis (Fig. 2B and C). The EdU proliferation assays showed that overexpression of circCPE significantly increased the number of EDU positive cells (Fig. 2D), which suggested that circCPE can promote DNA synthesis. The CCK8 assays revealed an increased vitality of myoblasts after overexpressing circCPE (Fig. 2E). In addition, we detected the cell cycle by flow cytometry assay and results showed that overexpression of circCPE increased the percentage of cells in the S phase and reduced cells at the G0/G1 phase and G2 phase (Fig. 2F).

Furthermore, two small interfering RNA (siRNA) targeted back-splicing junction was designed to silence circCPE. After transfected with siRNA-1 and siRNA-2 for 24 h, the expression of circCPE was reduced in bovine primary myoblasts, and we selected most effective siRNA-1 for subsequent experiments (Fig. S1A). The results indicated that the knockdown of circCPE significantly decreased the mRNA level of marker genes of *CDK2*, *cyclin D* and *cyclin E* and accompanied with decrease of its protein level (Fig. S1B and C). These results showed that the circCPE can promote proliferation of bovine myoblasts.

### CircCPE inhibited the apoptosis and differentiation of myoblasts

**Fig. 3:**
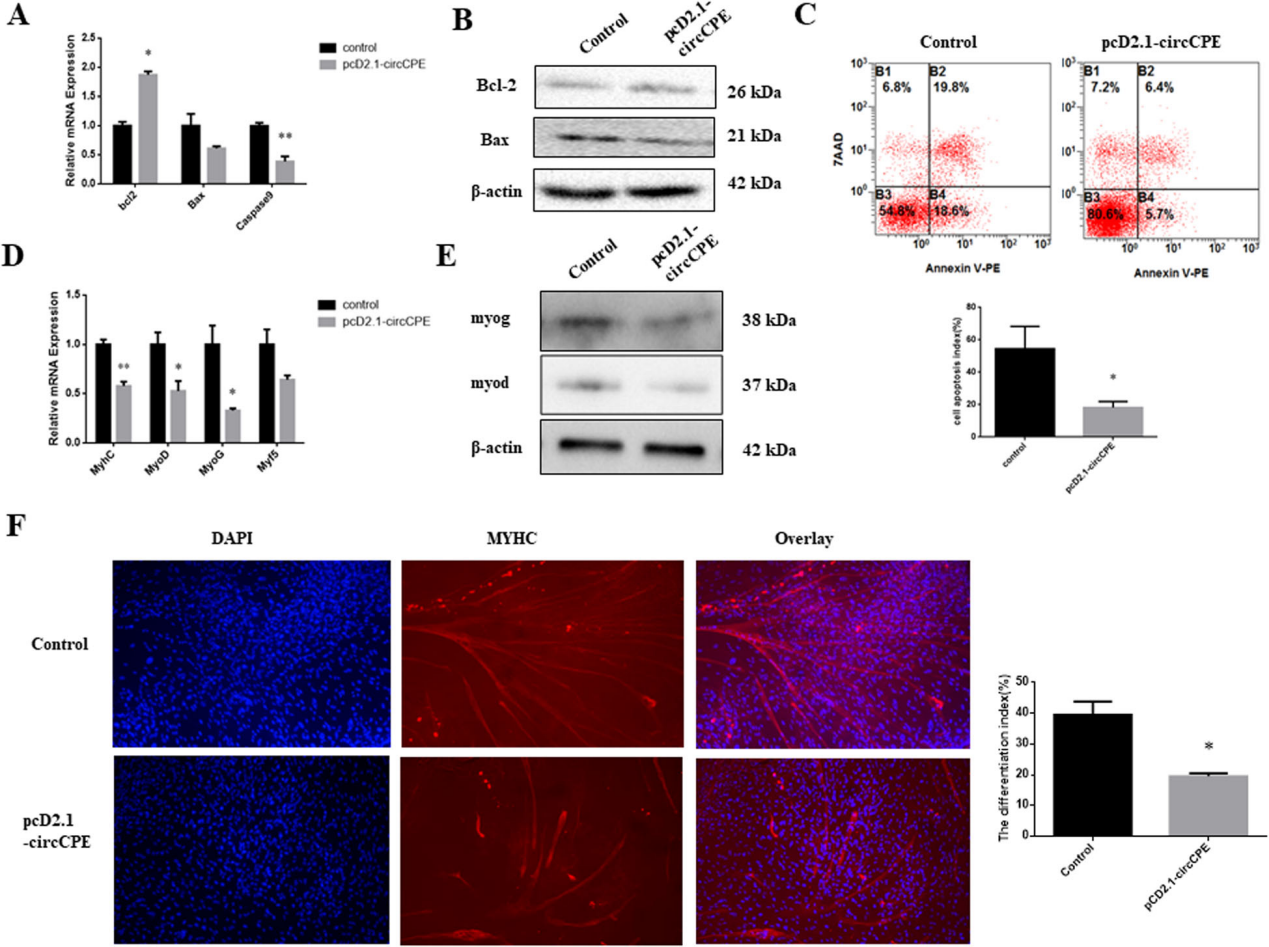
Overexpression of circCPE inhibited the apoptosis and differentiation of myoblasts. (**A** and **B**) The expression of apoptotic marker genes were detected using real-time qPCR and western blots after transfection with pcD2.1-circCPE vector. (**C**) Annexin V-APC/7AAD staining followed by flow cytometry revealed that circCPE inhibit myoblasts apoptosis. (**D** and **E**) The expression of differentiated marker genes was detected by qRT-PCR and western blot after transfection with pcD2.1-circCPE vector and culturing for 3 d. (**F**) Cell differentiation was detected by immunofluorescence (MyHC) and observed under a fluorescence microscope. Scale bar indicates 1,000 μm. Values are means ± SEM for three individuals. *P* < 0.05, *P* < 0.01

To investigate the potential functions of circCPE in myoblast apoptosis, we detected the marker genes of apoptosis by qRT-PCR and western blot analysis. The results showed that the overexpression of circCPE significantly reduced the expression of *Caspase9* and increased the expression of *Bcl2* at mRNA and protein level (Fig. 3A and B). There is a decline trend but the difference is not significant for *Bax* (Fig. 3B). Knockdown of circCPE resulted in promoting the expression of proapoptotic genes and inhibiting the expression of antiapoptotic gene in mRNA and protein level (Fig. S1D and E). Moreover, apoptosis assays using flow cytometry also demonstrated that circCPE have a restrained effect on myoblast apoptosis (Fig. 3C). Subsequently, we induced the myoblast differentiation to d 3 with 2% horse serum medium after transfection pCD2.1-circCPE for 24 h. As shown in Fig. 3D and E, the expression of marker genes of differentiation including *MyoG*, *MyoD*, and *MyHC* significantly reduced at mRNA and protein level (Fig. 3D and E). However, the level of *Myf5* had no significant differences (Fig. 3D). Inversely, the knockdown of circCPE significantly enhanced the expression of marker genes of differentiation at the mRNA level (Fig. S1F), as well as that of *MyoD* and *MyoG* proteins (Fig. S1G). Immunofluorescence assay also performed to evaluate whether circCPE affect myoblast differentiation. The results revealed that overexpression of circCPE inhibited the expression of *MyHC* as well as prevent the myotube formation (Fig. 3F). Additionally, expression of circCPE showed a sustained decline during myoblast differentiation (Fig. S2A and B). Consequently, our data suggested that circCPE inhibited the apoptosis and differentiation of bovine myoblasts.

### Overexpression of circCPE attenuates skeletal muscle regeneration

**Fig. 4:**
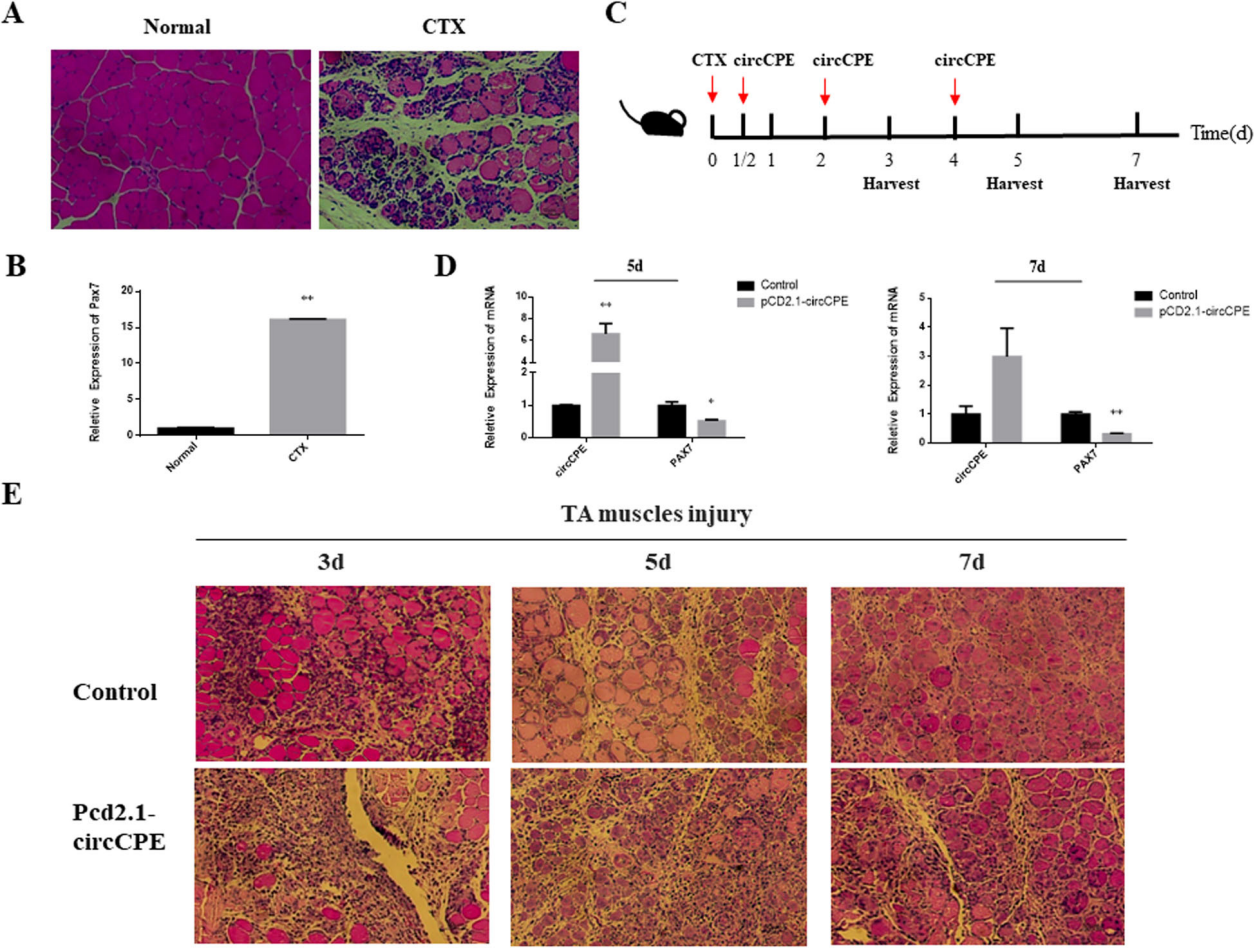
CircCPE attenuates skeletal muscle regeneration. (**A**) HE-staining of TA muscles after injection of CTX at 0 and 3 d. (**B**) The mRNA level of *Pax7* in TA after the injection of CTX. (**C**) Schematic representation of CTX injury followed by pCD-2.1-NC or pCD-2.1-circCPE treatment in mouse TA muscle. (**D**) The expression of circCPE and the changes of *Pax7* in TA muscle at 5 and 7 d, after CTX injury and plasmid injection. (**E**) HE-staining of CTX-injected muscle after transfection of the circCPE expression plasmid at 3, 5, and 7 d. Scale bar indicates 50 μm. Values are means ± SEM for three individuals. *P* < 0.05, *P* < 0.01

We further verified the function of circCPE in muscle development *in vivo* by building muscle injury model in mouse. The Hematoxylin and eosin (HE) staining showed that the mouse myofibers lysed to many scattered nuclei at the tibialis anterior (TA) muscle after Cardiotoxin (CTX) treatment (Fig. 4A). The mRNA levels of *Pax7*, a critical activation gene of satellite cell [28], were significantly increased in the CTX group compared with the normal group (Fig. 4B). These results indicated that the muscle damage model in mouse was successfully established. Subsequently, after CTX injection we transfected with circCPE overexpression plasmid on the half a day (1/2 d), 2 d and 4 d to experimentally maintaining circCPE long-term overexpression efficiency *in vivo* (Fig. 4C). Meanwhile, TA muscle was harvested to perform HE staining and qPCR at 3 d, 5 d and 7 d during muscle regeneration (Fig. 4C). The results revealed that the mRNA levels of circCPE were increased significantly on 5 d and 7 d, however the expression of *Pax7* were decreased in overexpression group than in the control (Fig. 4D). HE staining of TA sections showed that more muscle lysis has taken place in overexpression of circCPE at 3 d after CTX treatment, and at 5 d, 7 d newly formed small muscle bundles were less than in the control group (Fig. 4E). Therefore, our date demonstrated that overexpression of circCPE can attenuate TA muscle repair induced by CTX *in vivo*.

### CircCPE may serve as a miR-138 sponge

**Fig. 5:**
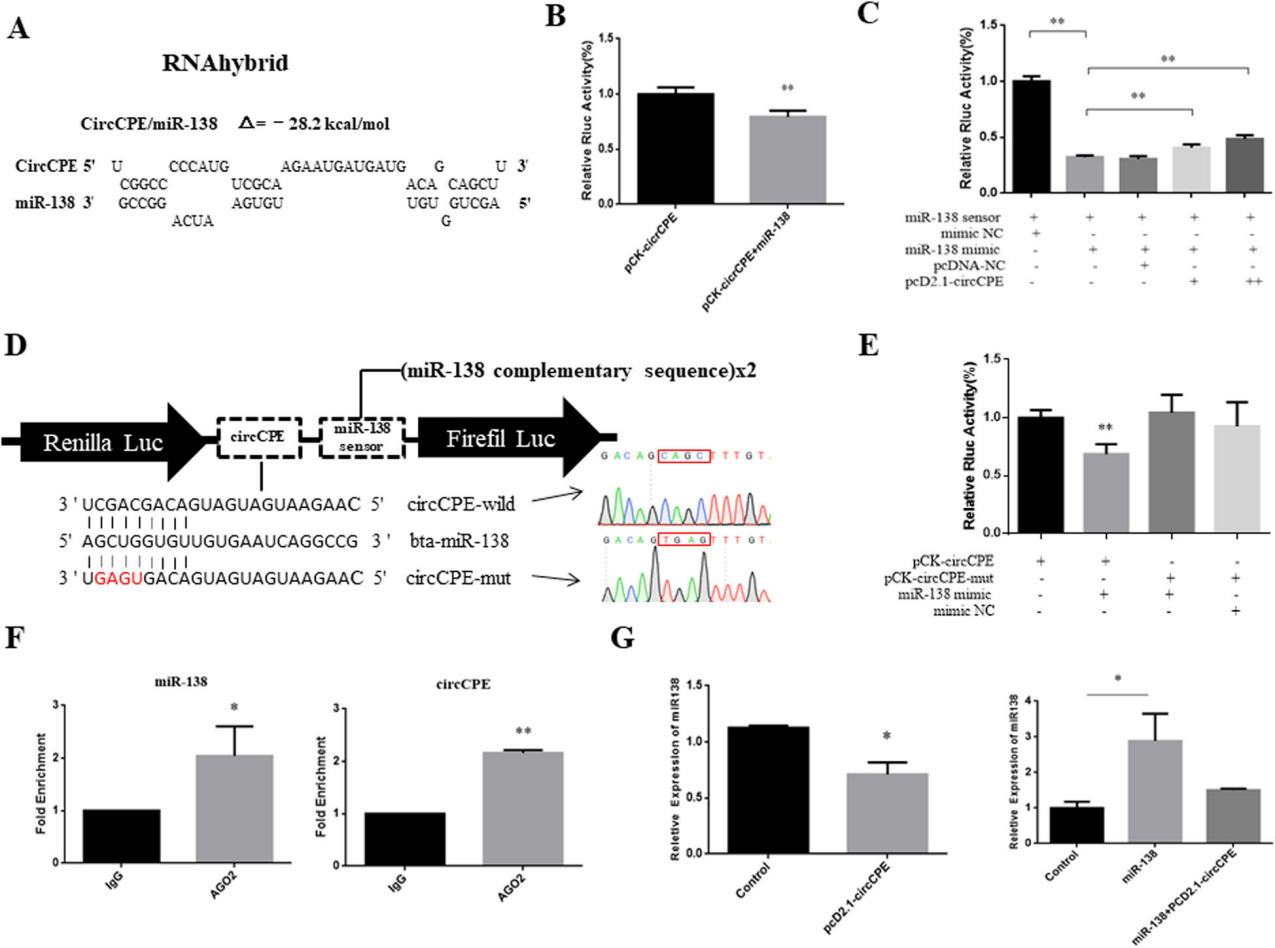
circCPE serves as a miR-138 sponge. (**A**) The proposed circCP binding site on miR-138 was identified using RNAhybrid. (**B**) The miR-138 mimic was co-transfected with pCK-circCPE into HEK293T cells. Luciferase activities were measured 24 h after transfection. Renilla luciferase activity was normalized to Firefly luciferase activity. (**C**) The miR-138 biosensor (psiCHECK2-miR-34a 2×) was co-transfected with the miR-138 mimic and 1× or 2× pcD2.1-circCPE into HEK293T cells, and luciferase activities were measured after transfection. (**D**) The illustration shows the construct of circCPE containing wild-type miR-138 binding sites or mutated sites and schematic diagram of the miR-138 sensor structure. (**E**) miR-138 mimics were co-transfected with psiCHECK2-circCPE-WT (pCK-circCPE) or psiCHECK2-circCPE-MUT (pCK-circCPE-mut) into HEK293T cells, and luciferase activities were measured after transfection. (**F**) Ago2-RIP assay for the amount of circCPE and miR-138 in bovine myoblasts. RIP experiments showed that the anti-AGO2 antibody efficiently captured circCPE and miR-138 transcripts. (**G**) Effects of overexpression with circCPE on the expression level of miR-138. Values are means ±SEM for three individuals. *P* < 0.05, *P* < 0.01

The most outstanding role of circRNAs is action as a miRNA sponge to regulate target gene expression [29]. Therefore, we next addressed the potential function as competing ceRNAs of circCPE in cytoplasm. To explore potentially targeted miRNA of circCPE, we screened a number of miRNAs involved in the skeletal muscle development and performed miRNA target sequence analysis using a complete fragment of circCPE through RNAhybrid bioinformatics program. We found that circCPE was potentially targeted by miR-138. The distribution of potential miR-138 binding sites is shown in Fig. 5A. MiR-138 has been reported affecting the various cancers cell proliferation and metastatic ability [30]. In addition, miR-138 is also involved in the proliferation, differentiation and apoptosis of smooth muscle cells [31, 32]. To test the miRNA sponge hypothesis, luciferase assay showed that miR-138 significantly inhibited Rluc activity of pCK-circCPE in HEK293T cells (Fig. 5B). Consistent results were observed that the Rluc activity of pCK-circCPE-mut had no responses on miR-138 (Fig. 5D and E). Subsequently, we constituted the miR-138 biosensor containing reverse complement repeats of miR-138 seed region (Fig. 5D), and co-transfection assay showed that overexpressing miR-138 significantly inhibited the Rluc expression of the biosensor. Importantly, Rluc activity was notably restored by co-transfection with pcD2.1-circCPE in a dose-dependent manner, which suggested that circCPE contain miR-138 binding sites and can adsorb miR-138 (Fig. 5C). To further confirm the sponge effect of circCPE, we also performed the Ago2 RNA immunoprecipitation (RIP) assay and results indicated that circCPE and miR-138 were significantly enriched by anti-Ago2 compared with anti-IgG (Fig. 5F). Additionally, the abundance of miR-138 was significantly reduced after overexpressing circCPE in bovine myoblasts, and the overexpression level of miR-138 also restored by co-transfection with pcD2.1-circCPE, which indicated that miR-138 can be adsorbed by circCPE (Fig. 5G). Taken together, these results indicated that circCPE acts as a sponge for miR-138.

### CircCPE regulated myoblasts proliferation, apoptosis and differentiation in a miR-138 dependent manner

**Fig. 6:**
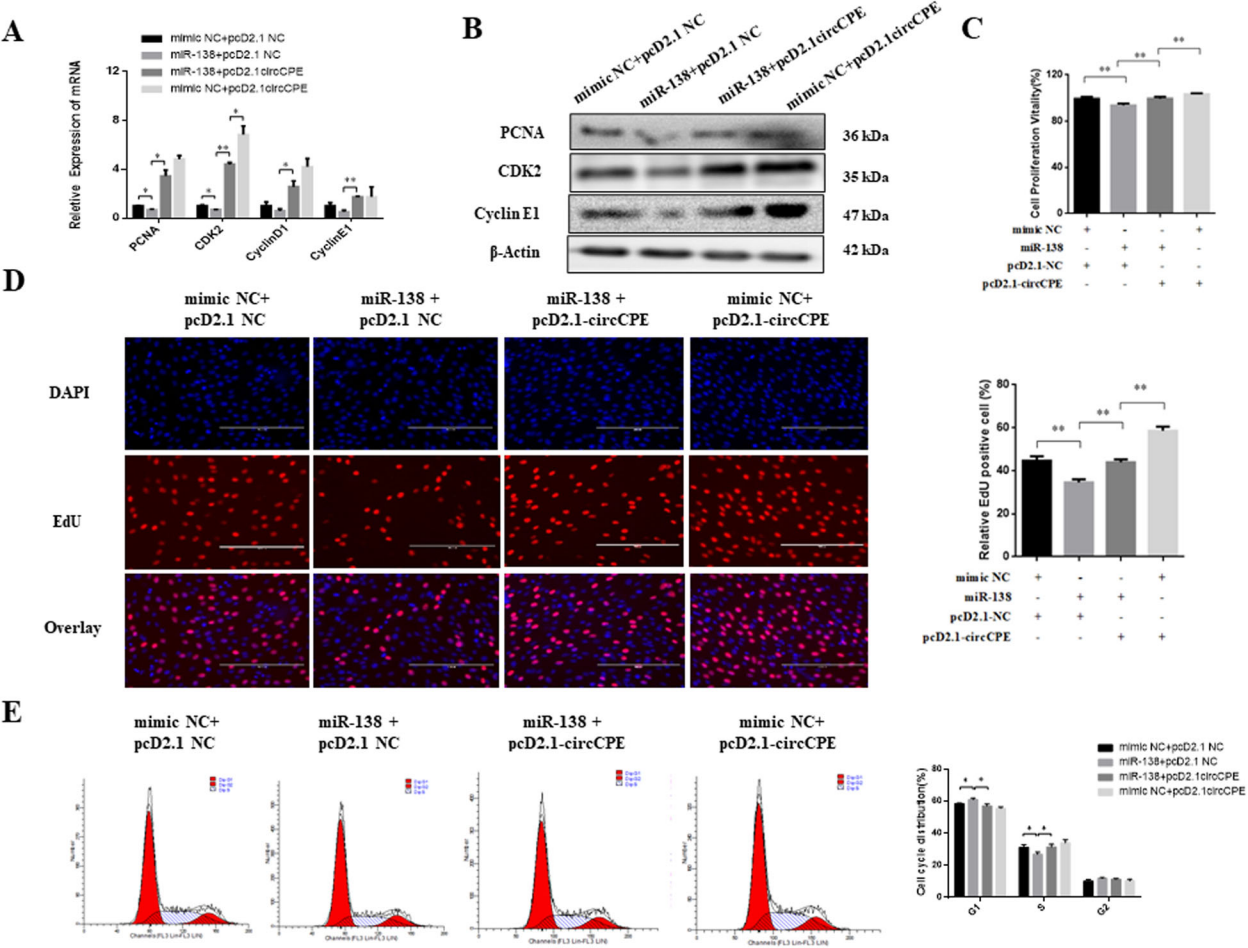
CircCPE promoted myoblasts proliferation in a miR-138 dependent manner. (**A** and **B**) The expression of marker genes of proliferation was detected by real-time qPCR and western blots after transfection with pCD2.1-NC + mimic NC, pCD2.1-NC + miR-138, pCD2.1- circCPE +miR-138 and pCD2.1-circCPE +mimic NC in bovine myoblasts. (**C** and **D**) The ability of proliferation was analyzed using EdU and CCK-8 after transfected with miR-138 mimics or co-transfected with circCPE or circCPE alone. (**E**) The cell cycle distribution was evaluated using flow cytometry assay after transfected with miR-138 mimics or co-transfected with circCPE or circCPE alone. Values are means ±SEM for three individuals. *P* < 0.05, *P* < 0.01

**Fig. 7:**
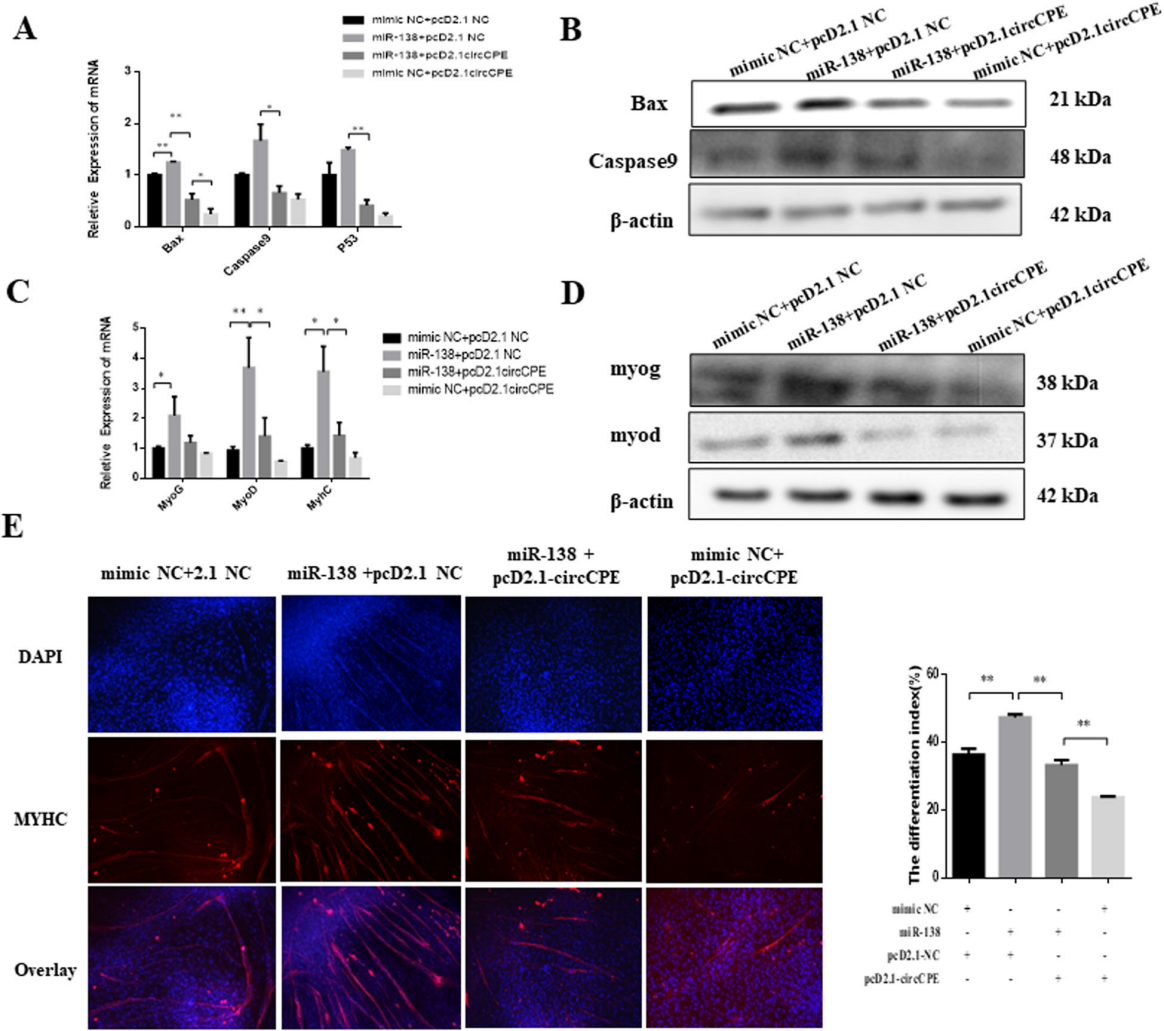
CircCPE inhibited myoblasts apoptosis and differentiation in a miR-138 dependent manner. (**A** and **B**) The expression of apoptosis marker genes was detected by real-time qPCR and western blots after transfection with pCD2.1-NC + mimic NC, pCD2.1-NC + miR-138, pCD2.1- circCPE +miR-138 and pCD2.1-circCPE +mimic NC in bovine myoblasts. (**C** and **D**) The expression of differentiated marker genes was detected by real-time qPCR and western blots after after transfection with pCD2.1-NC + mimic NC, pCD2.1-NC + miR-138, pCD2.1- circCPE +miR-138 and pCD2.1-circCPE +mimic NC in bovine myoblasts. (**E**) Cell differentiation was detected by immunofluorescence (MyHC) after transfected with miR-138 mimics or co-transfected with circCPE or circCPE alone. The images were observed under a fluorescence microscope (scale bar indicates 1000 μm). Values are means ± SEM for three individuals. *P* < 0.05, *P* < 0.01

On the basis of the interaction of circCPE and miR-138, we next performed the rescue experiments by co-transfecting pcD2.1-circCPE and miR-138 mimics in bovine myoblasts to evaluate whether the effect of miR-138 could be blocked by circCPE overexpression. The results of qRT-PCR and western blot analysis showed that miR-138 mimics significantly reduced the expression of marker genes of proliferation including *PCNA* and *CDK2*, but co-transfecting pcD2.1-circCPE remarkably relieve the miR-138 overexpressing-mediated inhibition of marker genes of proliferation (Fig. 6A and B). In addition, only overexpression of circCPE showed a higher stimulative proliferation than co-transfection pcD2.1-circCPE and miR-138 mimics (Fig. 6A and B). Similar changes were observed in CCK8 assays (Fig. 6C). The EdU proliferation assays also confirmed the elevated amount of EdU positive cells after co-transfection pcD2.1-circCPE and only transfection pcD2.1-circCPE compared with the group of miR-138 mimics (Fig. 6D). Moreover, cell cycle by flow cytometry assay also showed that the overexpression of circCPE counteracted the inhibitory effect of miR-138 on S phase, and promoted the transformation from G1 to S phase (Fig. 6E). To further assess the interaction of circCPE and miR-138 on myoblast apoptosis and differentiation, the results of qRT-PCR and western blot showed that the overexpression of circCPE can counteract the facilitation of miR-138 on the marker genes of apoptosis and differentiation (Fig. 7A-D). Meanwhile, the inhibiting effect of circCPE on apoptosis and differentiation was strengthen in group of transfecting pcD2.1-circCPE only (Fig. 7A-D). Immunofluorescence assay also demonstrated that the overexpression of circCPE blocked the enhancing effect of miR-138 on the expression of *MyHC* and myotube formation (Fig. 7E). As a consequence, circCPE can counteract the inhibitory effect of miR-138 on cell proliferation and the enhancing effect on apoptosis and differentiation.

### CircCPE serve as a miR-138 sponge to attenuate its inhibitory of *FOXC1*

**Fig. 8:**
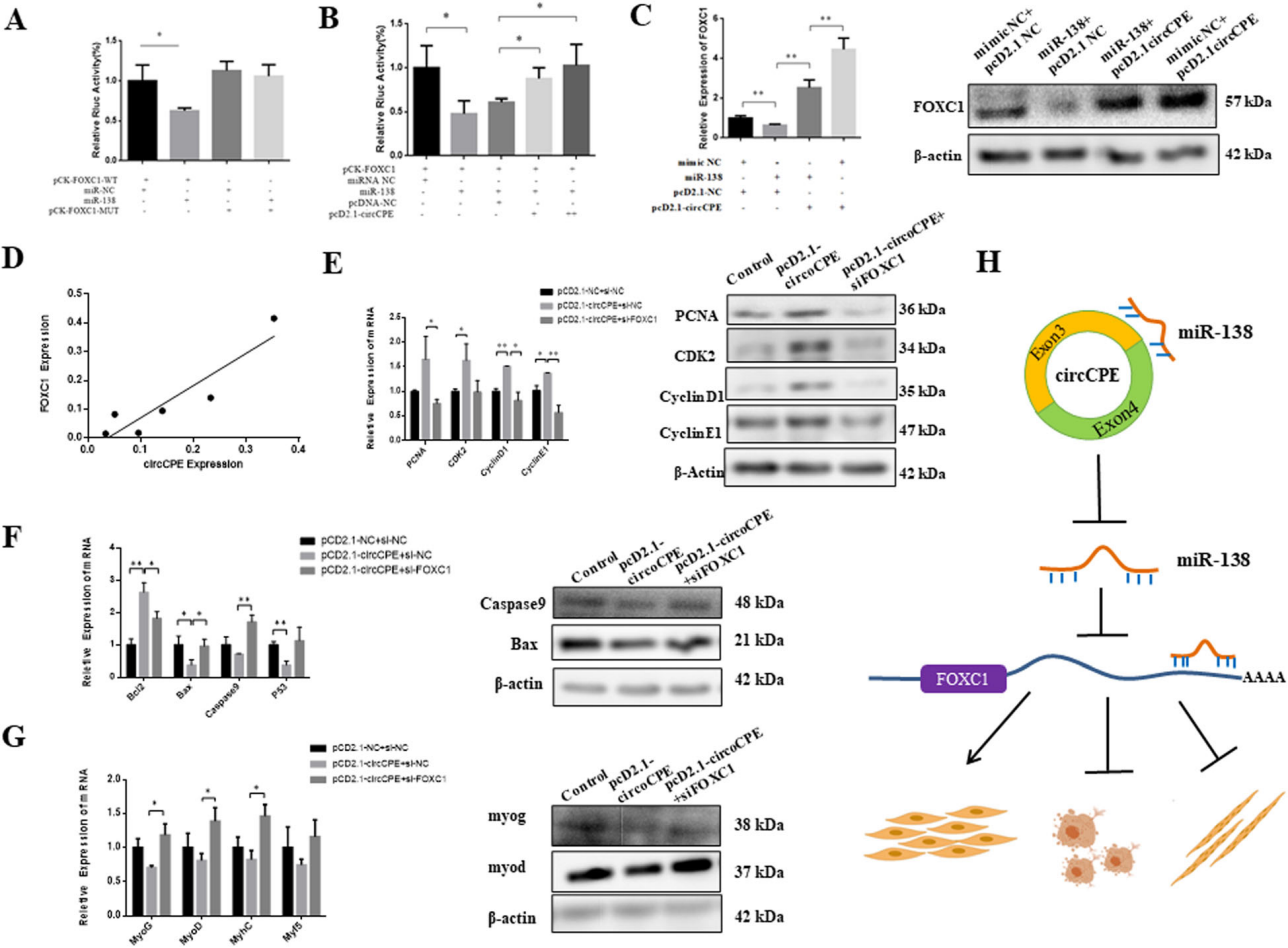
CircCPE serve as a mir-138 sponge to attenuate its inhibitory of *FOXC1*. (**A**) miR-138 mimics were co-transfected with pCK-*FOXC1*-WT or pCK- *FOXC1*- mut) into HEK293T cells. Luciferase activities were measured 24 h after transfection. Renilla luciferase activity was normalized to Firefly luciferase activity. (**B**) The pCK-*FOXC1*-WT was co-transfected with the miR-138 mimic and 1× or 2× pcD2.1-circCPE into HEK293T cells, and luciferase activities were measured after transfection. (**C**) The expression of *FOXC1* was detected by real-time qPCR and western blots after transfected with miR-138 mimics or co-transfected with circCPE or circCPE alone in bovine myoblasts. (**D**) The correlation analysis in expression of *FOXC1* and circCPE during myoblast differentiation (R^2^ = 0.84). (**E**) Effect of upregulated circCPE accompanied by suppressing *FOXC1* on the mRNA and protein levels of proliferation-related gene. (**F**) Effect of upregulated circCPE accompanied by suppressing *FOXC1* on the mRNA and protein levels of apoptosis maker genes. (**G**) Effect of upregulated circCPE accompanied by suppressing *FOXC1* on the mRNA and protein levels of differentiation marker genes. Values are means ± SEM for three individuals. *P* < 0.05, *P* < 0.01. (**H**) Schematic illustration of circCPE competitively binding miR-138 in regulating myoblast proliferation, apoptosis and differentiation

There are a number of studies reported that miR-138 in multiple cancer cells can block cell cycle arrest by targeting *cyclin D1* and *cyclin D3* [33, 34]. Our results also confirmed that the changes of *cyclin D1* at mRNA and protein are inversely correlated with upregulation of miR-138 in bovine myoblasts, which showed that *cyclin D1* acts as well-known targets of miR-138 (Fig. 6A). In addition, we used the bioinformatics software program TargetScan 7.2 to predict *FOXC1* also acting as target of miR-138. Next, we cloned the 3′ UTR (wild- and mutant-type) of *FOXC1* gene to the downstream of Renilla luciferase (Fig. S3A). The *FOXC1* 3′ UTR- wild activity was significantly suppressed by the overexpression of miR-138, but had no responses on *FOXC1* 3′ UTR- mutant (Fig. 8A). We also co-transfected miR-138 mimic and pcD2.1-circCPE, and found that Rluc activity was significantly restored by circCPE in a dose-dependent manner (Fig. 8B). Subsequently, the results of qRT-PCR and western blot analysis showed that miR-138 mimics significantly reduced the expression of *FOXC1*, but co-transfecting pcD2.1-circCPE remarkably increased the expression of *FOXC1,* which further consolidated the hypothesis that circCPE regulates gene expression as miR-138 sponge (Fig. 8C). Overexpression of circCPE intensely boosted *FOXC1* expression compared with co-transfection circCPE and miR-138 (Fig. 8C). It’s worth noting that there was a significant positive correlation between the *FOXC1* expression and circCPE expression during myoblast differentiation (Fig. 8D).

In addition, we also verified the function of *FOXC1* in bovine myoblasts development using a specific siRNA of *FOXC1* (*si-FOXC1*). The *si-FOXC1* was transfected in myoblasts and it successfully knockdown the expression of *FOXC1 *in mRNA and protein levels (Fig. S3B). For cells proliferation, the knockdown of *FOXC1* decreased the expression of proliferation-associated genes using qRT-PCR and western blot analysis (Fig. S3C and D). For cells apoptosis and differentiation, we found that the knockdown of *FOXC1* increased the expression of proapoptotic genes (Fig. S3E and F) and the expression of differentiation-related genes (Fig. S3G and H). These data suggested that *FOXC1* promoted proliferation and inhibited the apoptosis and differentiation in bovine myoblast, which consistent with the role of circCPE and inverse to that miR-138. Additionally, the results of qRT-PCR and western blot analysis showed that the depletion of *FOXC1* could reduce the facilitating effect of circCPE on cell proliferation (Fig. 8E). Meanwhile the depletion of *FOXC1* can also counteract the inhibiting effect of circCPE on cell apoptosis and differentiation (Fig. 8F and G). Taken together, all the results support the idea that circCPE acts as miR-138 sponge to protect the *FOXC1* from the attack of miR-138 thereby promoting cell proliferation and suppressing cell apoptosis and differentiation (Fig. 8H).

## Discussion

Although the biogenesis and function of circRNAs have been intensively investigated in the past few years, the role of circRNAs in myogenesis is largely unknown. In this study, we found that circCPE, a circRNA generated from second and third exons of carboxypeptidase E (CPE), is expressed differently in fetal and adult bovine muscle. *In vitro* studies indicated that circCPE possesses capability in promoting the bovine myoblast proliferation and inhibiting cell apoptosis and differentiation, and *in vivo* experiments revealed that overexpression of circCPE attenuates skeletal muscle regeneration. We then investigated the potential modes of circCPE on the regulatory functions in myogenesis. The results revealed that circCPE markedly suppressed the functions of miR-138 by capturing it and protect some genes from being attacked by miR-138. To our knowledge, this is the first report that researches the expression, function, and mechanism of circCPE in bovine muscle development.

Circular RNAs (circRNAs), a type of ncRNAs, generated by back-splicing and showed widespread, conserved, and tissue-specificity in mammals [29]. The lack of a 5′ cap and 3′ tail make the circRNAs more RNaseR resistance and longer half-lives compared to their linear transcript [35]. Although the number of the functional mechanism of circRNA studies has grown exponentially in past couple years, the most well-known role of circRNA is action as sponge to suppressing activity of miRNAs. Especially in the occurrence and progression of various diseases, the studies about interaction with circRNA and miRNA have been extensively described. For example, knockdown of human *CDR1as* result in dysregulating expression of miR-7 to affecting growth and metastasis of multiple cancer cells [36, 37]. Several recent studies have revealed that circRNAs as miRNA sponges participate in the muscle development. CircSVIL promotes the embryonic skeletal muscle development by sequestering miR-203 in chicken [38]. CircZfp609 can sponge miR-194-5p to repress the myogenic differentiation in mouse myoblast cell line (C2C12) [21]. Similarly, bovine circLMO7 acts as a decoy for miR-378a-3p promotes proliferation whereas inhibits apoptosis and differentiation of myoblast [27]; bovine circHUWE1 sponges the miR-29b-AKT3 axis to promote proliferation while inhibit apoptosis and differentiation of myoblasts [24]; and bovine circINSR promotes proliferation and inhibits apoptosis of myoblasts by sponging miR-34a [25].

Specifically, we observed a dramatic increase of circCPE expression in embryonic muscle tissue compared with adult muscle, suggesting a potential regulatory role in the muscle development. Sequencing verification, RNase R treatment and Actinomycin D treatment confirmed the circular nature of circCPE in bovine muscle. Functional assays showed that overexpression of circCPE could induce acceleration on proliferation and inhibition on apoptosis and differentiation. To further examine the functions of circCPE during myogenesis *in vivo*, we experimentally overexpressed circCPE in the TA muscle after CTX treatment. We found that circCPE had attenuate role in injury-induced muscle regeneration *in vivo*. Although the conservativeness of circCPE between mice and bovine is 86%, the miR-138 is highly conserved among species (Fig. S4). Therefore, we hypothesize that the exogenous circCPE plasmid could adsorb miR-138 in mouse muscles to restraining muscle regeneration. However, there are various models for circRNAS to play functions, and the regulatory mechanism of circCPE in vivo require further study.

A recent study discovered that circRNA can protect its parental transcript, such as circFOXO3 acts as a miRNA sponge to protect *FOXO3* mRNA from miRNA attack [39]. However, our study indicated that the expression of circCPE does not affect the level of the *CPE* mRNA in overexpression experiments, which implied that the regulation of *CPE* transcription and the formation of the circCPE are two independent events. FISH assay revealed circCPE abundant in the cytoplasm. In consideration of circRNA action as miRNAs sponge, our finding indicated that circCPE harbor miR-138 binding sites and absorbed miR-138.

MiR-138 has been identified as a signature of tumor suppressor that was found to express at low level across many human cancer types [30, 40]. Dysregulation on miR-138 can affect cancer cell proliferation, metastatic ability and apoptosis. Several studies have demonstrated the importance role of miR-138 in smooth muscle cell. MiR-138 can suppress airway smooth muscle cell and vascular smooth muscle cell proliferation [32, 41]. On the other hand, miR-138 has also been shown to function as an oncogenic miRNA in tumor-initiating glioma stem cells (GSC), which affects tumor recurrence and survival [42]. Interestingly, recent study found that miR-138 can accelerate the proliferation of human pulmonary artery smooth muscle cells (PASMCs) [31]. These findings indicate that the significant differences in tumor type and cell type are likely partly responsible for the function differences. In the present study, miR-138 was observed in inhibiting the myoblast proliferation and promoting apoptosis and differentiation. Luciferase reporter analysis confirmed that miR-138 could be adsorbed by circCPE. CircCPE functions as a sponge for miR-138 to counteract the inhibitory effect of miR-138 on cell proliferation and the enhancing effect on apoptosis and differentiation. Furthermore, only overexpression of circCPE showed a stronger stimulative proliferation and inhibiting apoptosis and differentiation than co-transfection circCPE and miR-138. The accumulating evidence has shown that miRNAs targeting the coding region was restricted by translation machinery [13]. Nevertheless, most circRNAs lack translation ability which may make circRNA as a better miRNA sponge compared with its linear transcript. It is not surprise that a circRNA sponges multiple miRNAs to regulate different genes. As with example, circHIPK3 increases *PTEN* and *RASA1* expression by targeting miR-17and miR-224, respectively [43]. However, whether circCPE possess multiple miRNAs binding sites remains to be further investigate.

As we all know, miR-138 can mediate multiple signaling pathways and various target genes to represses cell proliferation promote cell apoptosis [40]. Especially, miR-138 represses cell proliferation by targeted *cyclin D3 (CCND3)* and *cyclin D1 (CCND1),* as an important regulatory factor of cell cycle [33, 44]. Consistent results were also observed in our results. The changes of *cyclin D1* at mRNA are inversely correlated with upregulation of miR-138 in bovine myoblasts. Forkhead box C1 (*FOXC1*) was recently identified as a novel target of miR-138 [42]. *FOXC1* was identified as an important regulator of gene expression that plays a critical role in embryonic development [45]. It was later found to play a critical role in cancer cells invasion, proliferation and invasion, especially in studies of basal-like breast cancer (BLBC) [46]. *FOXC1* promotes BLBC cell growth, proliferation, and invasion through enhance the expression of the transcription factor *c-Myc* and *Cyclin D* [47]. In endometrial cancer, miR-204 and miR-495 can suppress the expression of *FOXC1* and caused in inhibiting cell growth and migration while increasing apoptosis [48, 49]. In addition, *FOXC1* could improve myocardial repair and restrain myocardial differentiation [50], and regulates early cardiomyogenesis [51]. In present study, we found that *FOXC1* promoted proliferation and inhibited the apoptosis and differentiation of bovine myoblast, which consistent with the role of circCPE and inverse to that miR-138. Meanwhile our results showed overexpression of miR-138 decreased the level of *FOXC1* on mRNA and protein. Luciferase reporter analysis also confirmed that *FOXC1* was a target gene of miR-138. CircCPE acting as miR-138 sponge suppressed the functions of miRNA accompanied with increased level of *FOXC1* as expected in our hypothesis. Notably, the depletion of *FOXC1* could reverse the regulatory effects of circCPE on the cell proliferation, apoptosis and differentiation.

Taken together, we firstly investigated the role and mechanism of circCPE in bovine skeletal muscle development. The results indicated that circCPE serves as a miR-138 sponge to protect *FOXC1* and resulted in promoting the bovine myoblast proliferation and inhibited cell apoptosis and differentiation *in vitro*. Moreover, *in vivo* experiments demonstrated that the inhibitory role of circCPE in repairing muscle damage. We constructed a novel circCPE/miR-138/*FOXC1* regulatory network in bovine myogenesis, which further provide evidence to illustrate a ceRNA function of circRNA in bovine muscle development. In addition, our results showed circCPE is also enrichment in the nucleus, so we still need to further explore the function of circCPE in the nucleus.

## Conclusions

Taken together, we firstly investigated the role and mechanism of circCPE in bovine skeletal muscle development. The results indicated that circCPE serves as a miR-138 sponge to protect *FOXC1* and resulted in promoting the bovine myoblast proliferation and inhibited cell apoptosis and differentiation i*n vitro*. Moreover, *in vivo* experiments revealed that overexpression of circCPE attenuates skeletal muscle regeneration. Based on the competitive endogenous RNA (ceRNA) theory, we constructed a novel circCPE/miR-138/*FOXC1* regulatory network in bovine myogenesis, which further provide evidence to illustrate a ceRNA function of circRNA in bovine muscle development. In addition, our results showed circCPE is also enrichment in the nucleus, so we still need to further explore the function of circCPE in the nucleus.

## Supplementary Information

**Additional file 1 Fig. S1.** Effect of circCPE knockdown on proliferation, apoptosis and differentiation of myoblasts. (**A**) The interference efficiency of the siRNAs to circCPE was detected by qRT-PCR. (**B** and **C**) The mRNA and protein level of proliferative marker genes was detected by qRT-PCR and western blot after transfection with si-circCPE-1. (**D** and **E**) The mRNA and protein level of apoptotic marker genes was detected by qRT-PCR and western blot after transfection with si-circCPE-1. (**F** and **G**) The expression of differentiated marker genes was detected by real-time qPCR and western blots after transfection with si-circCPE-1. Values are means ± SEM for three individuals. *P* < 0.05, *P* < 0.01

**Additional file 2 Fig. S2.** The expression of circCPE and *FOXC1* during myoblasts differentiation. (**A**) The picture illustration of bovine primary myoblast-induced proliferation and differentiation for − 1, 0, 1, 2, 3, and 4 d. (**B**) The expression changes of circCPE in bovine primary myoblasts proliferation and differentiation. (**C**) The expression changes of FOXC1 in bovine primary myoblasts proliferation and differentiation

**Additional file 3 Fig. S3.** Effect of *FOXC1* knockdown on proliferation, apoptosis and differentiation of myoblasts. (**A**) The illustration shows the construct of *FOXC1* 3′ UTR containing wild-type miR-138 binding sites or mutated sites. (**B**) The interference efficiency of the siRNAs to *FOXC1* was detected by qRT-PCR and western blot. (**C** and **D**) The mRNA and protein level of proliferative marker genes was detected by qRT-PCR and western blot after transfection with si-FOXC1. (**E** and **F**) The expression of apoptotic marker genes was detected by real-time qPCR and western blots after transfection with si-FOXC1. (**G** and **H**) The mRNA and protein level of differentiated marker genes was detected by qRT-PCR and western blot after transfection with si-FOXC1. Values are means ± SEM for three individuals. *P* < 0.05, *P* < 0.01

**Additional file 4 Fig. S4.** The conservation analysis of circCPE and miR-138. (**A**) The sequence alignment of circCPE between mice and bovine. (**B**) Conservation analysis of miR-138 among different species

**Additional file 5.**

**Additional file 6.**

## Data Availability

All data generated or analyzed during this study are included in this published article.

## Abbreviations

DAPI: 4′,6-diamidino-2-phenylindole
EdU: 5-ethynyl-2′ –deoxyuridine
CCK8: Cell Counting Kit-8
Rlu: Renilla luciferase
