# Supplementary material for: The circular RNA circCPE regulates myoblast development by sponging miR-138
Source: J Anim Sci Biotechnol. 2021 Sep 8;12:102. doi: 10.1186/s40104-021-00618-7 (PMC8424951; doi:10.1186/s40104-021-00618-7)
Supplement: Supplementary file 1 — Additional file 1 Fig. S1. Effect of circCPE knockdown on proliferation, apoptosis and differentiation of myoblasts. (A) The interference efficiency of the siRNAs to circCPE was detected by qRT-PCR. (B and C) The mRNA and protein level of proliferative marker genes was detected by qRT-PCR and western blot after transfection with si-circCPE-1. (D and E) The mRNA and protein level of apoptotic marker genes was detected by qRT-PCR and western blot after transfection with si-circCPE-1. (F and G) The expression of differentiated marker genes was detected by real-time qPCR and western blots after transfection with si-circCPE-1. Values are means ± SEM for three individuals. P < 0.05, P < 0.01 [file 40104_2021_618_MOESM1_ESM.docx]

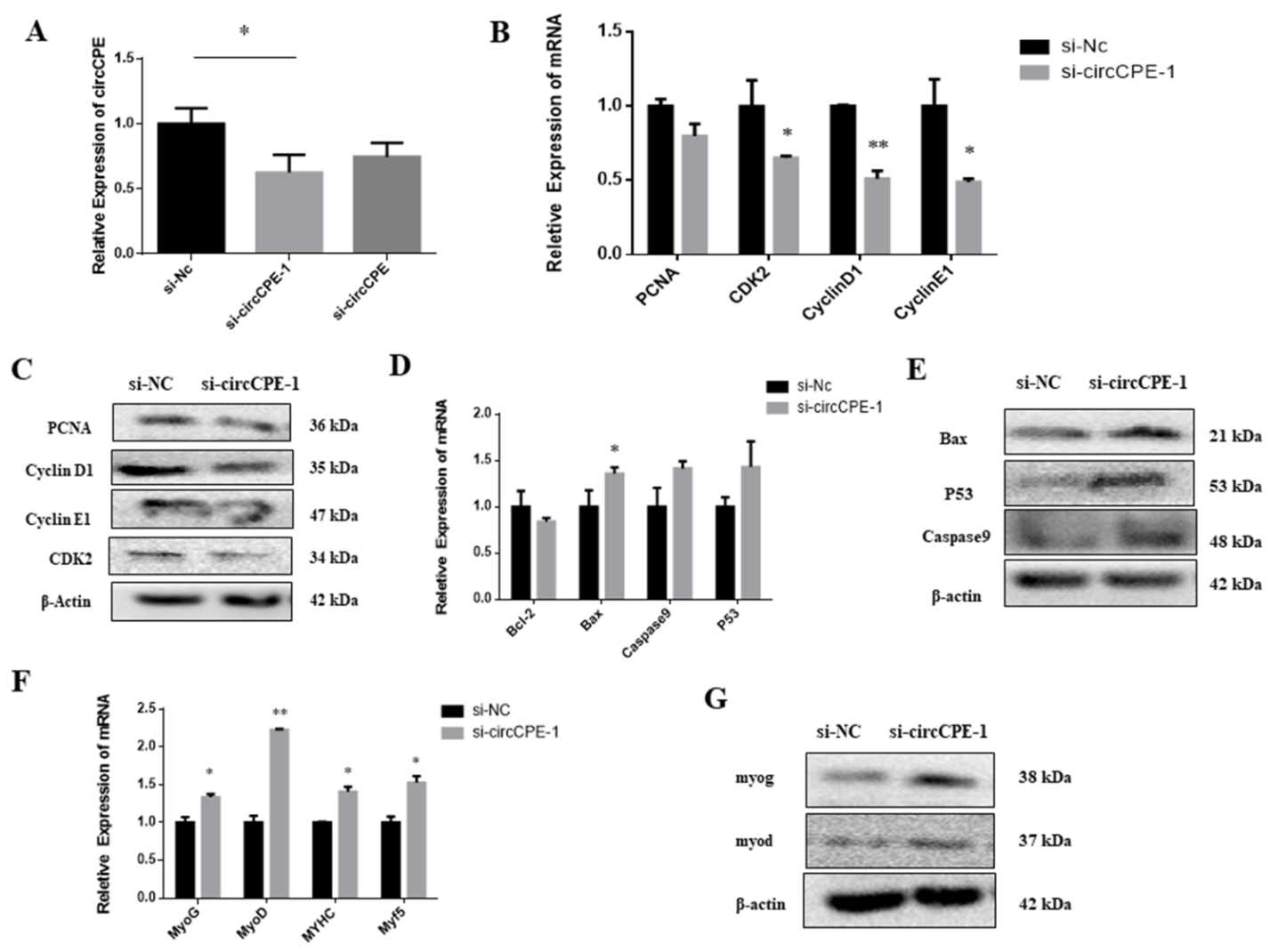


**Fig S1. Effect of circCPE knockdown on proliferation，apoptosis and differentiation of myoblasts.** (A) The interference efficiency of the siRNAs to circCPE was detected by qRT-PCR. (B and C) The mRNA and protein level of proliferative marker genes was detected by qRT-PCR and western blot after transfection with si-circCPE-1. (D and E) The mRNA and protein level of apoptotic marker genes was detected by qRT-PCR and western blot after transfection with si-circCPE-1. (F and G) The expression of differentiated marker genes was detected by real-time qPCR and western blots after transfection with si-circCPE-1. Values are means ± SEM for three individuals. *P* < 0.05, *P* < 0.01.
