# Supplementary material for: The circular RNA circCPE regulates myoblast development by sponging miR-138
Source: J Anim Sci Biotechnol. 2021 Sep 8;12:102. doi: 10.1186/s40104-021-00618-7 (PMC8424951; doi:10.1186/s40104-021-00618-7)
Supplement: Supplementary file 2 — Additional file 2 Fig. S2. The expression of circCPE and FOXC1 during myoblasts differentiation. (A) The picture illustration of bovine primary myoblast-induced proliferation and differentiation for − 1, 0, 1, 2, 3, and 4 d. (B) The expression changes of circCPE in bovine primary myoblasts proliferation and differentiation. (C) The expression changes of FOXC1 in bovine primary myoblasts proliferation and differentiation [file 40104_2021_618_MOESM2_ESM.docx]

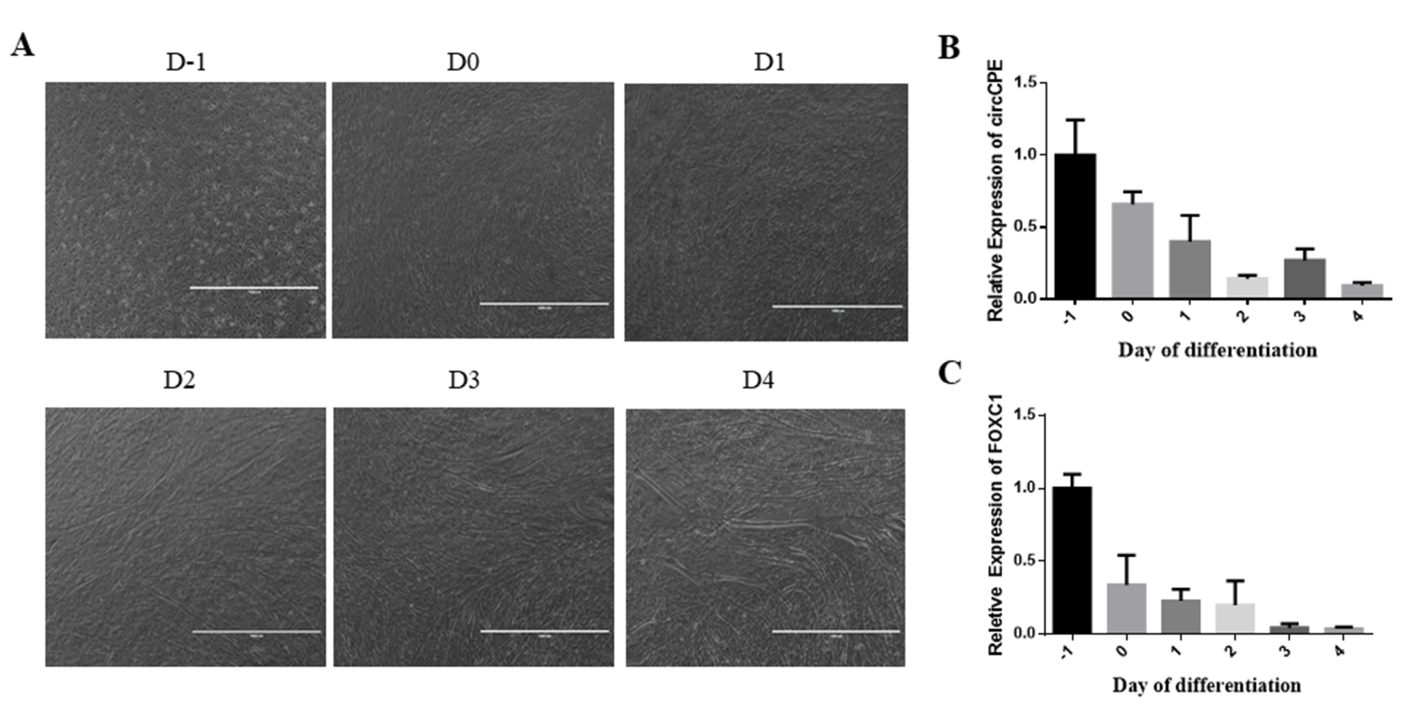


**Fig S2. The expression of circCPE and FOXC1 during myoblasts differentiation.** (A) The picture illustration of bovine primary myoblast-induced proliferation and differentiation for -1, 0, 1, 2, 3, and 4 d. (B) The expression changes of circCPE in bovine primary myoblasts proliferation and differentiation. (C) The expression changes of FOXC1 in bovine primary myoblasts proliferation and differentiation.
