# Supplementary material for: The circular RNA circCPE regulates myoblast development by sponging miR-138
Source: J Anim Sci Biotechnol. 2021 Sep 8;12:102. doi: 10.1186/s40104-021-00618-7 (PMC8424951; doi:10.1186/s40104-021-00618-7)
Supplement: Supplementary file 3 — Additional file 3 Fig. S3. Effect of FOXC1 knockdown on proliferation, apoptosis and differentiation of myoblasts. (A) The illustration shows the construct of FOXC1 3′ UTR containing wild-type miR-138 binding sites or mutated sites. (B) The interference efficiency of the siRNAs to FOXC1 was detected by qRT-PCR and western blot. (C and D) The mRNA and protein level of proliferative marker genes was detected by qRT-PCR and western blot after transfection with si-FOXC1. (E and F) The expression of apoptotic marker genes was detected by real-time qPCR and western blots after transfection with si-FOXC1. (G and H) The mRNA and protein level of differentiated marker genes was detected by qRT-PCR and western blot after transfection with si-FOXC1. Values are means ± SEM for three individuals. P < 0.05, P < 0.01 [file 40104_2021_618_MOESM3_ESM.docx]

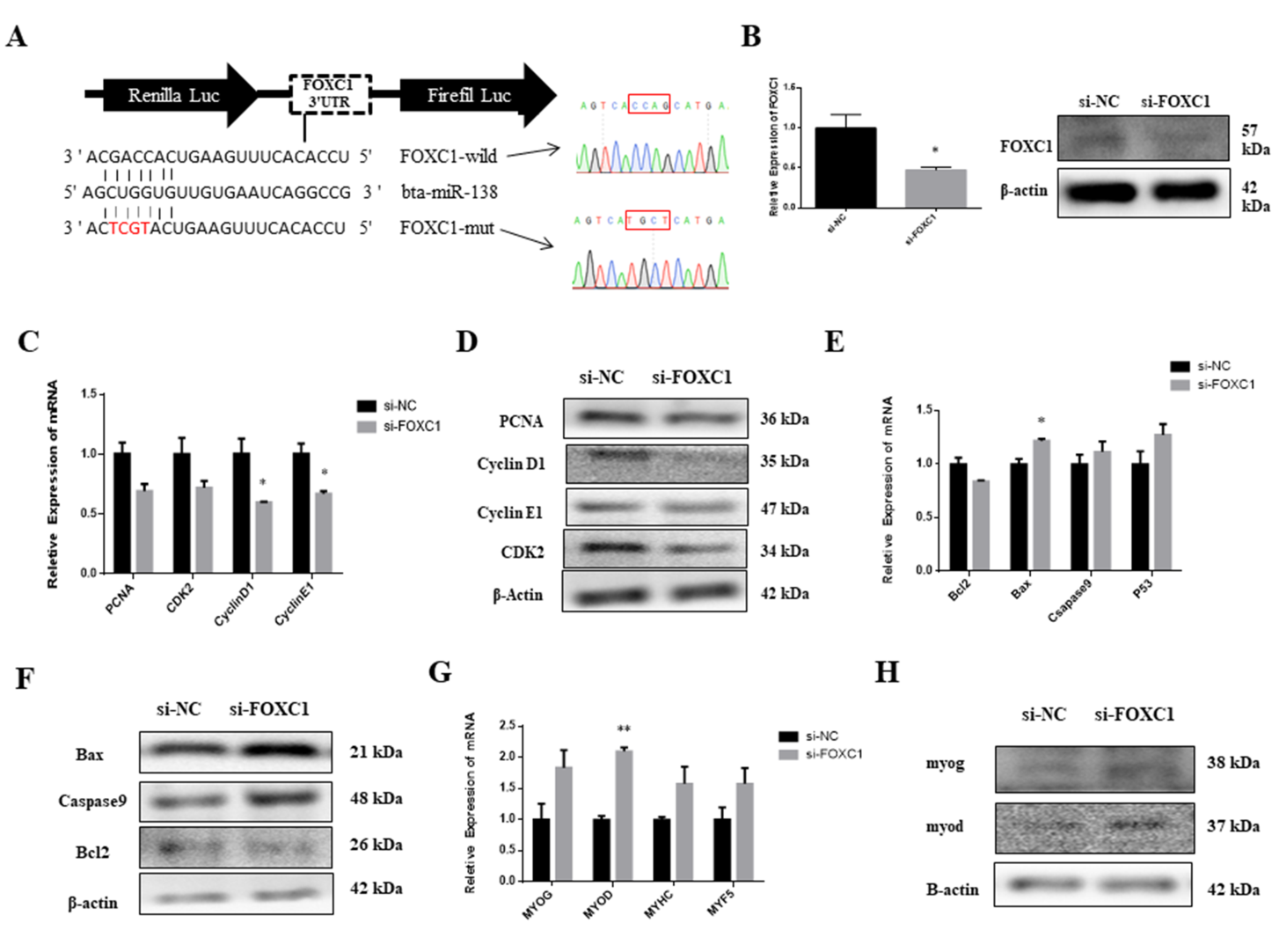


**Fig S3.** **Effect of *FOXC1* knockdown on proliferation，apoptosis and differentiation of myoblasts.** (A) The illustration shows the construct of *FOXC1* 3´ UTR containing wild-type miR-138 binding sites or mutated sites. (B) The interference efficiency of the siRNAs to *FOXC1* was detected by qRT-PCR and western blot. (C and D) The mRNA and protein level of proliferative marker genes was detected by qRT-PCR and western blot after transfection with si-FOXC1. (E and F) The expression of apoptotic marker genes was detected by real-time qPCR and western blots after transfection with si-FOXC1. (G and H) The mRNA and protein level of differentiated marker genes was detected by qRT-PCR and western blot after transfection with si-FOXC1. Values are means ± SEM for three individuals. *P* < 0.05, *P* < 0.01.
