# Supplementary material for: The circular RNA circCPE regulates myoblast development by sponging miR-138
Source: J Anim Sci Biotechnol. 2021 Sep 8;12:102. doi: 10.1186/s40104-021-00618-7 (PMC8424951; doi:10.1186/s40104-021-00618-7)
Supplement: Supplementary file 4 — Additional file 4 Fig. S4. The conservation analysis of circCPE and miR-138. (A) The sequence alignment of circCPE between mice and bovine. (B) Conservation analysis of miR-138 among different species [file 40104_2021_618_MOESM4_ESM.docx]

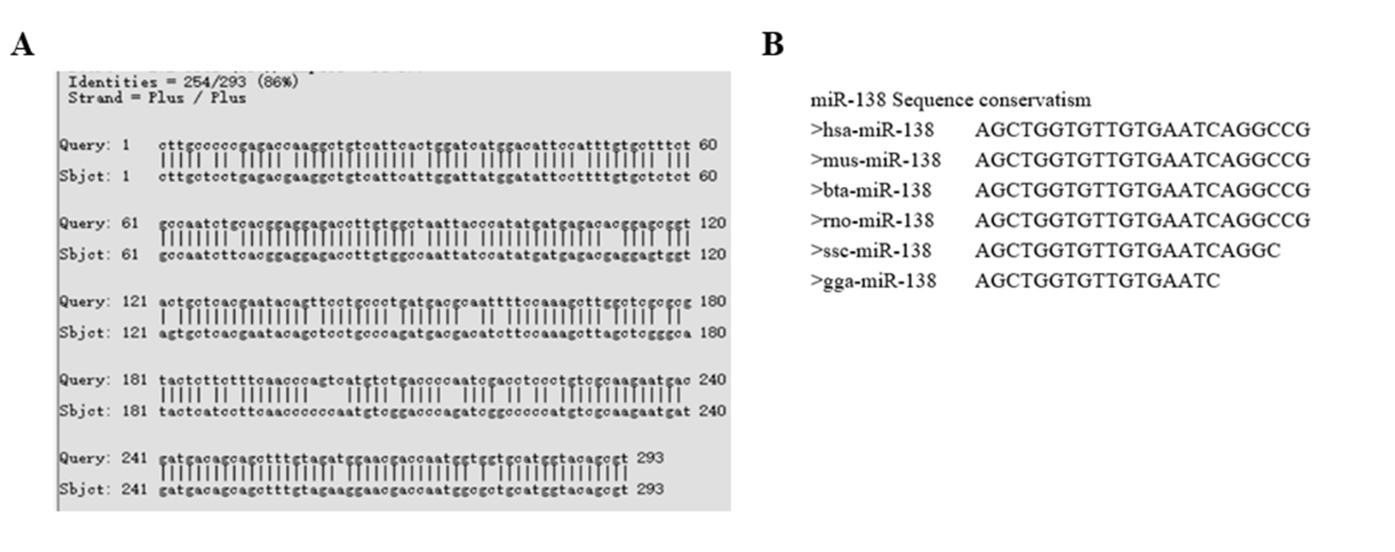


**Fig S4. The conservation analysis of circCPE and miR-138.** (A) The sequence alignment of circCPE between mice and bovine. (B) Conservation analysis of miR-138 among different species.
