# Supplementary material for: The circular RNA circCPE regulates myoblast development by sponging miR-138
Source: J Anim Sci Biotechnol. 2021 Sep 8;12:102. doi: 10.1186/s40104-021-00618-7 (PMC8424951; doi:10.1186/s40104-021-00618-7)
Supplement: Supplementary file 5 — Additional file 5. [file 40104_2021_618_MOESM5_ESM.doc]

Supplementary table S1：Primer Information in our work

| Divergent Primer-F | CCCCCATGTCGCAAGAATGA |
| --- | --- |
| Divergent Primer-R | AGCACTACCACTCCTCGTCT |
| Convergent Primer-F | AGACGAGGAGTGGTAGTGCT |
| Convergent Primer-R | GATCTGGGTCCGACATTGGG |
| pcD2.1-circCPE-F | GGGGTACCCTTGCTCCTGAGACGAAGGC |
| pcD2.1-circCPE-R | CGGGATCCCTCCAGGCACGCTGTACCAT |
| pCK-circCPE-mut-F | CCGCTCGAGCTTGCTCCTGAGACGAAGGC |
| pCK-circCPE-mut-R | TTGCGGCCGCCCTTCTACAAACTCACTGTCATCATCA |
| miR-138-sensor-F | TCGAGCGGCCTGATTCACAACACCAGCTCGGCCTGATTCACAACACCAGCTGC |
| miR-138-sensor-R | GGCCGCAGCTGGTGTTGTGAATCAGGCCGAGCTGGTGTTGTGAATCAGGCCGC |
| miR-138-RT | GTCGTATCCAGTGCAGGGTCCGAGGTATTCGCACTGGATACGACCGGCCTGA |
| miR-138-F | TGCGCGAGCTGGTGTTGTG |
| miR-138-R | GTGCAGGGTCCGAGGT |
| GAPDH-F | CACTGAGGACCAGGTTGTCT |
| GAPDH-R | TGTCGTACCAGGAAATGAGC |
| β-actin-F | GTCATCACCATCGGCAATGAG |
| β-actin-R | AATGCCGCAGGATTCCATG |
| U6-F | GCTTCGGCAGCACATATACTAAAAT |
| U6-R | CGCTTCACGAATTTGCGTGTCAT |
| PCNA-F | AACCTCACCAGCATGTCCAA |
| PCNA-R | CCAACGTGTCCGCGTTATCT |
| CDK2-F | TCTTTGCTGAGATGGTGACCC |
| CDK2-R | CATCTTCATCCAGGGGAGGC |
| Cyclin E1-F | CGATGTCTCTGTTCGCTCCA |
| Cyclin E1-R | CCACACTGGCTTCTCACAGT |
| Cyclin D1-F | ATGAAGGAGACCATCCCCCT |
| Cyclin D1-R | CGCCAGGTTCCACTTGAGTT |
| P21-F | AGGGCACGTCTCAGGAGGA |
| P21-R | CAGTCTGCGTTTGGAGTGGTAG |
| BCL2-F | ATGACCGAGTACCTGAAC |
| BCL2-R | CATACAGCTCCACAAAGG |
| BAX-F | GAGATGAATTGGACAGTAACA |
| BAX-R | TTGAAGTTGCCGTCAGAA |
| P53-F | CCTCCCAGAAGACCTACCCT |
| P53-R | CTCCGTCATGTGCTCCAACT |
| MYOD-F | AACACTACAGCGGCGACTC |
| MYOD-R | GCTGTAGTCCATCATGCCGT |
| MYOG-F | CCAGTACATAGAGCGCCTGC |
| MYOG-R | AGATGATCCCCTGGGTTGGG |
| MYHC-F | TGCTCATCTCACCAAGTTCC |
| MYHC-R | CACTCTTCACTCTCATGGACC |
| MYF5-F | TCTATCTCTCTGCTGTCCAGGC |
| MYF5-R | GTACTCAGAGGGCGAGAACTG |
| PAX7-F | CTGCTGAAGGACGGTCACTG |
| PAX7-R | GGATGCCATCGATGCTGTGT |
| FOXC1-F | ATGTTTGAGTCGCAGCGGA |
| FOXC1-R | AGAACTTGCTGCAGTCGTAGA |
| Pck-FOXC1-WT-F | CCGCTCGAGCCAGTGCGTAAAACGTCCAC |
| Pck-FOXC1-WT-R | TTGCGGCCGCCACTTTCTGGCGTTTGGTCC |
| Pck-FOXC1-mut-F | CCGCTCGAGCCACACTTTGAAGTCATGCTCATGAAGAAAAGGCT |
| Pck-FOXC1-mut-R | TTGCGGCCGC CACTTTCTGGCGTTTGGTCC |
