# Supplementary material for: The circular RNA circCPE regulates myoblast development by sponging miR-138
Source: J Anim Sci Biotechnol. 2021 Sep 8;12:102. doi: 10.1186/s40104-021-00618-7 (PMC8424951; doi:10.1186/s40104-021-00618-7)
Supplement: Supplementary file 6 — Additional file 6. [file 40104_2021_618_MOESM6_ESM.docx]

>circCPE

CTTGCTCCTGAGACGAAGGCTGTCATTCATTGGATTATGGATATTCCTTTTGTGCTCTCTGCCAATCTTCACGGAGGAGACCTTGTGGCCAATTATCCATATGATGAGACGAGGAGTGGTAGTGCTCACGAATACAGCTCCTGCCCAGATGACGACATCTTCCAAAGCTTAGCTCGGGCATACTCATCCTTCAACCCCCCAATGTCGGACCCAGATCGGCCCCCATGTCGCAAGAATGATGATGACAGCAGCTTTGTAGAAGGAACGACCAATGGCGCTGCATGGTACAGCGTGCCTGGAG
